# Editorial: Three-way interactions between host, environment, and microbiome: Importance of microbiology in the One Health

**DOI:** 10.3389/fmicb.2023.1177119

**Published:** 2023-03-21

**Authors:** Caihong Wu, Ruixin Zhu, Yongxu Lu, Dong Li, Yuan Xiao, Weiwei Cui, Na Li

**Affiliations:** ^1^Department of Nutrition and Food Hygiene, School of Public Health, Jilin University, Changchun, China; ^2^Department of Bioinformatics, School of Life Sciences and Technology, Tongji University, Shanghai, China; ^3^Department of Pathology, University of Cambridge, Cambridge, United Kingdom; ^4^Department of Immunology, College of Basic Medical Sciences, Jilin University, Changchun, China; ^5^Department of Pediatrics, Ruijin Hospital, Shanghai Jiao Tong University School of Medicine, Shanghai, China; ^6^School of Tropical Medicine, The Second Affiliated Hospital, Hainan Medical University, Haikou, China

**Keywords:** microbiome, host health and disease, environmental, interactions, One Health

The microbiome is burgeoning as one of the new frontiers in the field of biomedical research. The potential of research is as colossal and varied as the microbes in these communities. Microbes such as viruses, bacteria, prions, and fungi as disease vectors have been intensively studied in recent years, contributing to great advances in medicine. As research on gut microbes has progressed, microbes have generally come to be considered as closely related to hosts and as having a vital role in maintaining human health, ranging from digestive functions to nutrient uptake, and regulating the immune response. However, little research has examined the role of microbes in host health in certain environments. There is a huge knowledge gap as to how the microorganisms, environment, and host interact with each other and what the results are.

Microbiology continues to attract the attention of ecologists and medical scientists, resulting in the stimulation of numerous initiatives to study the effects of the microbiome on host health in different microenvironments. In this Research Topic, “*Three-way interactions between host, environment, and microbiome: Importance of microbiology in the One Health*,” a total of nine articles were published, covering a variety of topics that involve a variety of microbes in different microenvironments, including bacteria in the tumor microenvironment, the microbiota of different anatomical sites (nasopharyngeal, oropharyngeal, lung, and gut), and the discovery of novel, bioactive functional substances such as a microsatellite DNA-derived oligodeoxynucleotide, hypothetical protein FoDbp40, and *Stenotrophomonas maltophilia*. Moreover, the synergy of the microbiome was reported, focusing on the effects of the regulation of intestinal flora in conjunction with drug use on host health.

In recent times, research on the microbiome has evolved from the culture of intestinal and oral microbiota to a mechanistic comprehension of the host–microbiome connection and a microbiome map of all the niches in the body. Xu et al. discussed the diversity and specificity of bacteria in different cancers. The amount and multiformity of bacteria in breast tumor samples were greater than in normal breast samples. In contrast, the microbiome of lung cancer tissue changed less than that of the corresponding normal tissue. A particular bacterium, *Helicobacter pylori*, has been associated with stomach cancer and specified by the World Health Organization as a class I carcinogen; however, no conclusive results on the potential role of other bacterial species in most cancer types have been reported. Many challenges remain in the identification of tumor-specific bacteria. Xu et al. further reviewed the connection between macrophages and bacteria in cancer. Bacteria may multiply in macrophages and control them *via* a variety of interference tactics. Macrophage polarization seems to be an intermediate procedure activated by some signals during tumorigenesis and regression. Macrophages show diverse phenotypes in response to multiple stimuli that act on various receptors and thus play a regulatory role through multiple signaling pathways. Chen et al. also focused on microbiomes at different anatomical sites. Chen et al. analyzed the gut and respiratory tract (oropharynx, nasopharynx, and lung) microflora of normal and influenza A virus (IAV)-infected mice and compared the microflora structure on diverse mucosal surfaces. It was shown that the pulmonary flora of healthy mice was mainly nasopharyngeal, and IAV infection could increase the microbiota similarity between the lungs and nasopharynx. Importantly, *Lactobacillus murinus* was identified as a biomarker for IAV infection, because its abundance was decreased in all ecological niches.

*Fusarium oxysporum*, a fungus existing in the environment that can infect crops such as cotton, rice, and wheat, is also an important opportunistic pathogen of humans, which seriously affects food safety and human health (Kazan and Gardiner, 2018; Alkatan and Al-Essa, 2019; Zhu et al., 2021). The study of Zhao et al. indicated that the gene encoding isocitrate lyase is a pivotal element that affects the growth of *Fusarium oxysporum*, and FoDbp40 protein could regulate the activity of isocitrate lyase, affect the ATP level and the AMPK/mTOR pathways, and consequently regulate *Fusarium oxysporum* virulence and growth. Dai et al. reviewed various toll-like receptors (TLRs) that are involved in the immunopathogenesis of severe acute respiratory syndrome coronavirus 2 (SARS-CoV-2) infection. Dai et al. made clear the potential application value of the application of TLR immunomodulators in patients with coronavirus disease 2019 (COVID-19). Some viruses and fungi living in the natural environment, and likewise environmental factors such as smoking and exposure to chemicals, may cause changes in the composition and structure of an organism's microbiome that can affect host health. Shen et al. demonstrated an observable augmentation of the genus *Stenotrophomonas* in lung adenocarcinoma (LADC) tissues of non-smoking patients with a primary tumor size greater than 3 cm. Shen et al. further found that *Stenotrophomonas* treatment drove inflammation and upregulated tumor-associated cell signaling in the nitrosamines 4-(methylnitrosamino)-1-(3-pyridyl)-1-butanone-induced lung cancer mouse model. In addition, histone deacetylase 5 gene expression was significantly upregulated in *Stenotrophomonas*-treated groups and was required for *Stenotrophomonas*-induced cell proliferation and migration in LADC cell line A549. Zhang et al. previously designed an oligodeoxynucleotide (named MS19) with six AAAG repeated units based on human microsatellite DNA sequences. Zhang et al. demonstrated that MS19 inhibited pathogen-associated molecular patterns-induced inflammatory responses *in vitro* and *in vivo*. This inhibition is related to nuclear factor kappa B signal transduction but not to mitogen-activated protein kinase transduction. Moreover, Li et al. found that guanylate-binding protein 5 (GBP5) is highly expressed in the colonic immune cells of inflammatory bowel disease (IBD) patients. Induction of GBP5 is required for the stimulated production of proinflammatory cytokines and chemokines, while GBP5 deficiency decreases the expression of the proinflammatory mediators. These results suggest that targeting GBP5 may be an effective strategy for IBD management.

Exposure to radiation, heat and cold stimulation, animal fur, pollen, chemicals, parasites, and other aspects of the environment may cause human skin inflammation and even skin diseases. However, it is not clear whether the progression of skin diseases is connected with the changes in human microbes. Liu et al. analyzed changes of the intestinal flora in stool samples from melasma patients and healthy subjects by 16S rRNA sequencing, observing that many significantly different microbiomes are closely related to beta-glucuronidase production and the regulation of estrogen metabolism. These findings imply that changes of the gut microbiota structure in melasma patients can play an important role in the occurrence and development of melasma by affecting the body's estrogen metabolism.

Either Shugan decoction (SGD) or fecal microbiota transplantation (FMT) can alleviate the symptoms of irritable bowel syndrome (IBS) in patients and animal models. However, the synergistic effect of FMT and SGD on IBS symptoms is not clear. Meng et al. conducted relevant research and found that SGD and FMT had no synergistic effect on water avoidance stress (WAS)-induced IBS model rats. It was suggested that the metabolites of intestinal microbiota may be the main active substances of the FML derived from normal rats to alleviate WAS-induced IBS symptoms.

Taken together, these studies provide crucial insights into the interactions between the microbiota change, environment, and host health and emphasize the potential of a microsatellite DNA-derived oligodeoxynucleotide, hypothetical protein FoDbp40, and *Stenotrophomonas maltophilia*. However, the mechanisms behind these phenomena need to be further studied. Moreover, future research should also focus on the role of extreme environmental conditions, such as extreme temperatures and ultraviolet radiation, in shaping the multiniche microbiome and its consequence to host health. Overall, the studies have provided increasing evidence on the importance of the microbiome and highlighted the need for further research into the interactions between the host, environment, and microbiome.

## Author contributions

All authors listed have made a substantial, direct, and intellectual contribution to the work and approved it for publication.
